# Response to influenza vaccination in immunocompromised children with rheumatic disease: a prospective cohort study

**DOI:** 10.1186/s12969-021-00518-0

**Published:** 2021-03-12

**Authors:** Lotte Jensen, Susan Nielsen, Anne Estmann Christensen, Freddy Karup Pedersen, Ramona Trebbien, Thea Kølsen Fischer, Susanne Rosthøj, Peter Toftedal, Anna-Helene Bohr, Peder Skov Wehner, Anja Poulsen

**Affiliations:** 1 The Department of Paediatrics and Adolescent Medicine, Juliane Marie Centre, Rigshospitalet, Copenhagen University Hospital, Copenhagen, Denmark; 2 Faculty of Health and Medical Sciences, University of Copenhagen, Copenhagen, Denmark; 3 The Department of Paediatrics and Adolescent Medicine, Hans Christian Andersen Children’s Hospital, Odense University Hospital, Odense, Denmark; 4 Department of Virus and Microbiological Special diagnostics, National Influenza Center, Statens Serum Institut, Copenhagen, Denmark; 5 Section of Biostatistics, Department of Public Health, University of Copenhagen, Copenhagen, Denmark; 6 The Department of Paediatrics, Naestved Hospital, Naestved, Denmark

**Keywords:** Paediatric, Biological DMARDs, Immunization, Influenza, Antibody, Rheumatic

## Abstract

**Background:**

Prevention of illness due to infection by influenza viruses is important for children with rheumatic diseases. Biological disease modifying antirheumatic drugs have become increasingly important in the treatment of juvenile idiopathic arthritis, and combinations of immunosuppressive drugs are used for the treatment of systemic disorders, which increase the risk of secondary immunodeficiency. Therefore, we investigated whether children with rheumatic disease can mount a protective antibody response after influenza immunization.

**Methods:**

The prospective multicentre cohort study was conducted in Denmark during the influenza season 2015–2016. Children with rheumatic disease aged six months to 19 years were eligible. Controls were immunologically healthy children. A blood sample was collected before and after vaccination and analysed by haemagglutination inhibition (HI) assay for the 2015–2016 influenza vaccine-strains. In case of flu-like symptoms the child was tested for influenza. For statistical analyses the patients were grouped according to medical treatment or disease.

**Results:**

A total of 226 patients and 15 controls were enrolled. No differences were found for the increase of antibodies from pre-vaccine to post-vaccine between the groups in our primary analyses: A/Cal H1N1pdm09 (*p* = 0.28), A/Swi H3N2 (*p* = 0.15) and B/Phu Yamagata (*p* = 0.08). Only when combining patients across groups a lower increase in antibodies was found compared to controls. Among all patients the pre-vaccine rates for seroprotection using the HI-titer cut-off ≥ 40 were 93.1–97.0 % for all three strains. For seroprotection using the HI-titer cut-off ≥ 110 the pre-vaccine rates for all patients were 14.9–43.6 % for all three strains and an increase in the proportions of patients being seroprotected after vaccination was found for A/Cal H1N1pdm09 and A/Swi H3N2. None of the children with flu-like symptoms tested positive for the vaccine strains.

**Conclusions:**

Children with rheumatic diseases increase in antibody titres after influenza immunization, however, it remains uncertain whether a protective level is achieved.

## Background

Immunization is considered an effective preventive measure in reducing the risk of illness due to infection by influenza viruses. For children with juvenile idiopathic arthritis (JIA) and systemic connective tissue disorders prevention of infection is important, as both their underlying disease and often long-term use of immunosuppressive treatment increase the risk of a severe course of infection [1, 2]. Therefore, for high risk groups such as immunocompromised individuals immunization including annual seasonal influenza vaccine is recommended [3, 4]. The existing evidence for response to influenza vaccination in children with rheumatic diseases is primarily based on studies where patients are treated with combinations of corticosteroids and disease modifying antirheumatic drugs (DMARDs) [5, 6] or where only a limited number of patients are treated with biological DMARDs (bDMARDs) [7–9]. As bDMARDs have become increasingly important in the treatment during the last decades [10], we estimate that half of the 1,200 paediatric JIA patients in Denmark are treated with bDMARDs [11]. More knowledge is needed for paediatric patients with rheumatic diseases about the response to influenza vaccination in terms of achieving seroprotection and being clinically protected.

## Materials and Methods

### Objective

The objective of this study was to compare the antibody response to influenza vaccine and laboratory confirmed influenza illness in children with rheumatic diseases and healthy controls.

### Study design and participants

Participants were prospectively recruited from a regional hospital and two tertiary referral hospitals in Denmark from September 2015 to January 2016. Inclusion criteria for all participants were age 6 months to 19 years and vaccination against seasonal influenza 2015–2016. Patients were suffering from a rheumatic disease and were vaccinated due to compromised immune system assessed by the clinician given the disease itself or the medical treatment. Controls were immunologically healthy children who were not taking any medication influencing the immune system, and they were vaccinated due to a chronic illness or as sibling to a child in treatment for cancer.

The patients were grouped according to medical treatment or disease as follows:

‘bDMARDs monotherapy’: Patients receiving bDMARDs except Rituximab;

‘bDMARDs + DMARDs’: Patients receiving bDMARDs (except Rituximab) and DMARDs;

‘Rituximab’: Patients receiving Rituximab at any time within the last six months before vaccination and possibly additional immunosuppressive medicine;

‘Systemic disorders’: Patients with systemic connective tissue disorders independent of medical treatment;

‘Other’: Patients with rheumatic diseases (except systemic connective tissue disorders) receiving immunosuppressive medications in combinations different from previous mentioned groups.

### Vaccination

Participants were vaccinated according to the guidelines for seasonal influenza from the Danish Health Authority and the Danish Paediatric Society, which recommend vaccination to risk groups [12, 13]. Children aged 6 months to 8 years not previously vaccinated against influenza were vaccinated twice with an interval of at least 4 weeks. Children aged 9 years and above, and children previously vaccinated against influenza, were only vaccinated once. The injection was administered intramuscularly in the deltoid.

The vaccines used were: Fluarix®, GlaxoSmithKline, Australia or Vaxigrip®, Sanofi, France. According to the WHO recommendation for the northern hemisphere in 2015–2016 the vaccines included A/California/07/2009-like (H1N1)pdm09 (A/Cal H1N1pdm09); A/Switzerland/9,715,293/2013-like (H3N2) (A/Swi H3N2); and B/Phuket/3073/2013-like (Yamagata-lineage) (B/Phu Yamagata) [14].

### Serological testing

The pre-vaccine blood sample was collected before vaccination or up to 3 days after vaccination [15]. The post-vaccine blood sample was collected as close to day 28 as possible, but up to day 120 post vaccination, at the first following routine blood sampling. The haemagglutination inhibition (HI) assay was used to test for the presence of antibodies against the influenza virus strains contained in the vaccine. Analyses were performed at the National Influenza Center, Statens Serum Institut, Copenhagen, Denmark according to the techniques described by WHO [16].

A selection of samples was also tested for functional antibodies by microneutralisation (MN) assay as described by WHO [16] but with the following modifications; influenza positive cells were immunostained with 3,3′-Diaminobenzidine (DAB) (Kem-En-Tec, diagnostics 4170) followed by manual inspection in microscope.

### Immunogenicity

Vaccination immunogenicity parameters were based on the European Medicines Evaluation Agency/Committee for Proprietary Medical Products (EMEA/CPMP) 1997 criteria for HI-assays [17]: Seroconversion was defined as negative pre-vaccine serum with post-vaccine serum ≥ 40; or a ≥ 4 fold rise in antibody titre [17]. There is no clear definition for seroprotection in children and therefore both a titre of ≥ 40 which is an estimate for 50 % clinical protection in adults [18, 19], and a titre of ≥ 110 which is an estimate for 50 % clinical protection in children aged 6 months to 6 years [20] were used.

### Flu‐like symptoms

If the child got flu-like symptoms a questionnaire addressing clinical symptoms was to be completed and a nasal swab was performed at home. Upon inclusion the family was taught how to use the nasal swab, and test kit and a questionnaire were handed over. After each self-reported episode of flu-like symptoms the family was sent a new test kit and questionnaire.

Nasal swab was performed using Universal Transport Media kit 3 ml, UTM™, COPAN. Viral RNA was extracted using 200 µL of sample material and the MagNA Pure LC Total Nucleic Acid Isolation Kit on the MagNapure 96/32 (Roche). Quantitative multiplex reverse transcriptase polymerase chain reaction qRT-PCR was used for detection and subtyping of influenza A and B viruses using the MX3005P Stratagene platform. Analyses were performed at the National Influenza Center, Statens Serum Institut, Copenhagen, Denmark, using in house designed assays.

### Statistical analysis

Quantitative data are presented as median values with inter quartile range (IQR) or as geometric means titre (GMT) with 95 % confidence intervals (95 % CI). Categorical data are presented as counts (%). To compare the groups with respect to the differences in pre-vaccine versus post-vaccine antibody titres for A/Cal H1N1pdm09, A/Swi H3N2, respectively B/Phu Yamagata, linear mixed effects models were used. The antibody levels were analysed on the log scale. The group and the timing (pre/post) were considered fixed effects and a random intercept was included for each participant. To investigate whether the increase in antibody titres differed between the groups a test of interaction between group and timing was performed. Due to low power of this test (because of the large number of degrees of freedom), an additional test of interaction between all patients as a group/controls and timing was performed. The analyses were adjusted for the number of days between vaccination and post vaccination blood sampling as fixed effect due to an imbalance in the timing between the groups and the linearity was assessed using linear splines with knots at day 60 and 120. To examine the effect of age (in groups 6 months to 11 years and 12–19 years), sex, duration of treatment with bDMARDs, and time from last injection of bDMARDs to vaccination, the analyses were repeated including these variables as fixed effects.

To compare the proportion of participants with seroprotection pre-vaccine to post-vaccine, the McNemar test was performed for all three influenza strains, for each group separately as well as for all patients. The analyses could not be performed when one of the proportions were 100 %. For each outcome and each cut off, the p-values for each group were adjusted for multiple testing using the Bonferroni method.

Data was analysed using SAS Enterprise Guide version 7.1.

## Results

**Fig. 1:**
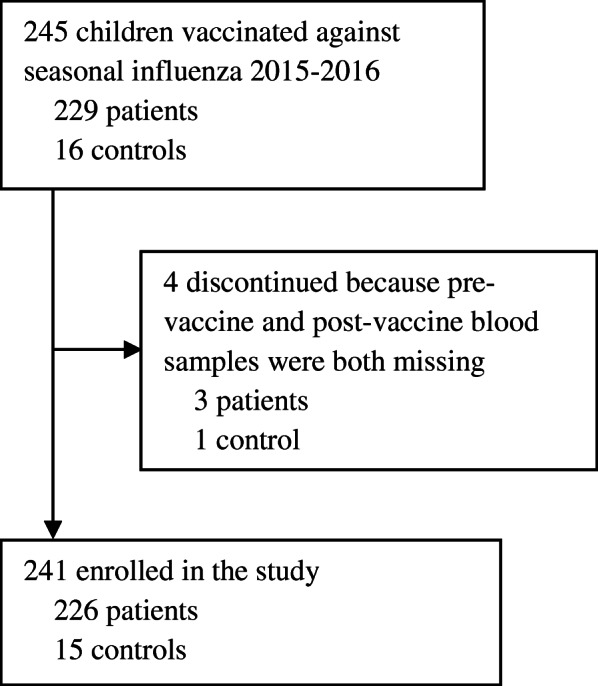
Flow-chart of participants in the study

A total of 245 children were eligible for inclusion and 226 patients and 15 controls were enrolled (Fig. 1). Median age for patients and controls were 14.2 years and 12.2 years, respectively. Baseline characteristics are shown in Table 1. The reasons for vaccination among controls were: Spinal muscular atrophy (*n* = 1), spherocytosis (*n* = 4), and healthy sibling to a child in treatment for cancer (*n* = 10).

**Table 1:** Characteristics of 226 patients and 15 controls vaccinated against seasonal influenza 2015–2016

Characteristics	All patients (*n* = 226)	bDMARDs monotherapy^a^ (*n* = 80)	bDMARDs + DMARDs ^b^ (*n* = 110)	Rituximab^c^ (*n* = 5)	Systemic disorders^d^ (*n* = 18)	Other^e^ (*n* = 13)	Controls (*n* = 15)
Age, years	14.2 (10.8–17.1)	14.1 (10.1–17.2)	13.7 (11.2–16.7)	15.3 (14.6–16.4)	15.9 (12.3–16.9)	17.2 (14.5–18.4)	12.2 (11.0-15.5)
Male sex	78 (34.5)	30 (37.5)	36 (32.7)	0	6 (33.3)	6 (46.2)	9 (60.0)
Age at onset of symptoms	7.8 (3.2–11.0)	7.1 (2.9–9.5)	7.0 (2.8–11.0)	11.3 (10.1–12.5)	11.0 (8.8–14.3)	9.6 (6.3–11.5)	-
Age at diagnosis	9.5 (4.4–12.8)	8.0 (3.4–11.9)	9.5 (5.3–12.7)	12.8 (10.1–13.4)	11.9 (10.0-14.8)	10.3 (7.8–12.2)	-
JIA – ILAR criteria							
Systemic arthritis	7	3	2			2	
Oligoarthritis	67	35	31			1	
Polyarthritis (RF-negative)	81	27	50	1		3	
Polyarthritis (RF-positive)	5	1	4				
Psoriatic arthritis	14	3	10			1	
Enthesitis-related arthritis	11	4	6			1	
Undifferentiated arthritis							
Systemic connective tissue disorders							
Granulomatosis with polyangiitis	4			1	3		
Juvenile dermatomyositis	4				4		
Mixed connective tissue disease	4				4		
Polymyositis	2				2		
Scleroderma	3				3		
Sjogren syndrome	1			1			
Systemic lupus erythematosus	3			2	1		
Other mixed connective tissue syndrome	1				1		
Other							
Behchet’s disease	1		1				
Chronic recurrent multifocal osteomyelitis	11	7	3			1	
Familial Mediterranean fever	4		1			3	
Iridocyclitis	2		1			1	
Sarcoidosis	1		1				
Duration of bDMARDs therapy, years	1.1 (0.4-2.0)	1.3 (0.3–2.3)	0.9 (0.4–1.8)			0.5 (0-0.7)	
Duration from last bDMARDs injection to vaccination, days	5.0 (2.0-11.5)	5.0 (2.0–9.0)	5.0 (3.0–14.0)			5.5 (0.0–10.0)	

Data are median (interquartile range) or No. (%)

Abbreviations: *DMARDs* disease modifying antirheumatic drugs. *bDMARDs* biological DMARDs. *ILAR* International League of Associations for Rheumatology. *JIA* Juvenile idiopathic arthritis. *RF* Rheumatoid factor

^a^ bDMARDs used: TNFα inhibitors 87.5 %, costimulation modulator (inhibits T lymphocyte)1,25 %, IL-6 inhibitor 11.25 %

^b^ bDMARDs used: TNFα inhibitors 87.4 %, costimulation modulator (inhibits T lymphocyte) 4.5 %, IL-1 inhibitor 0.9 %, IL-6 inhibitor 8.1 %. DMARDS used: Methotrexate 94.5 %, leflunomide 3.6 %, mycophenolate mofetil 0.9 %, colchicine 0.9 %, methotrexate + colchicine 0.9 %

^c^ Medication in addition to rituximab: glucocorticoid + immunoglobulin + mycophenolate mofetil 20 %, glucocorticoid + hydroxychloroquine + mycophenolate mofetil 40 %, glucocorticoid + hydroxychloroquine + mycophenolate mofetil + glucocorticoid bolus 20 %, leflunomide 20 %

^d^ Medication: glucocorticoid + mycophenolate mofetil + hydroxychloroquine 27.8 %, glucocorticoid + mycophenolate mofetil 11.1 %, glucocorticoid + immunoglobulin + methotrexate 5.6 %, mycophenolate mofetil 16.7 %, methotrexate 27.8 %, hydroxychloroquine 5.6 %, no medication 5.6 %

^e^ Medication: glucocorticoid bolus + TNFα inhibitor + glucocorticoid + methotrexate 7.7 %, glucocorticoid bolus + IL-6 inhibitor + glucocorticoid + methotrexate 7.7 %, glucocorticoid bolus + TNFα inhibitor + methotrexate 7.7 %, glucocorticoid bolus + TNFα inhibitor + mycophenolate mofetil 7.7 %, TNFα inhibitor + glucocorticoid + methotrexate 15.4 %, IL-6 inhibitor + glucocorticoid + methotrexate 7.7 %, IL-1 inhibitor + glucocorticoid 7.7 %, TNFα inhibitor + glucocorticoid + mycophenolate mofetil 7.7 %, glucocorticoid + leflunomide 7.7 %, no medication 23.1 %

The GMTs for the influenza strains contained in the 2015–2016 vaccine for patients and controls are shown in Table 2. Two patients had the pre-vaccine blood sample drawn one day after vaccination. The patients had the post-vaccine blood sample taken later (median 65 days, range 28–120) than controls (median 35 days, range 28–59) and as this 30-day difference in median time from vaccination to post-vaccine blood sampling was found to influence the level of the antibody titres, the increase in antibody level was adjusted for the number of days between vaccination and post-vaccination blood sampling. No differences were found in the increase of antibodies from pre-vaccine to post-vaccine between the groups: A/Cal H1N1pdm09 (*p* = 0.28), A/Swi H3N2 (*p* = 0.15) and B/Phu Yamagata (*p* = 0.08) (Table 3). Combining all the patients across groups into one group and comparing the differences between all patients and controls, we found that the percentage increase in antibodies was lower for patients compared to controls for A/Swi H3N2 with 74.9 % versus 193.2 % at day 28 post vaccination (*p* = 0.02), whereas no differences were found for A/Cal H1N1pdm09 with 62.7 % versus 74.2 % (*p* = 0.80); or B/Phu Yamagata with 12.3 % versus 53.9 % (*p* = 0.06) at day 28 post vaccination.

**Table 2:** Geometric mean titer (GMT) for influenza strains pre-vaccine and post-vaccine

	All patients (*n* = 226) (missing^a^: 24/28)	bDMARDs monotherapy (*n* = 80) (missing^a^: 8/14)	bDMARDs + DMARDs (*n* = 110) (missing^a^: 13/11)	Rituximab (*n* = 5) (missing^a^: 1/0)	Systemic disorders (*n* = 18) (missing^a^: 1/1)	Other (*n* = 13) (missing^a^: 1/2)	Controls (*n* = 15) (missing^a^: 4/2)
A/Cal H1N1pdm09							
Pre-vaccine	102.4 (92.1–113.9)	110.9 (91.8–134.1)	94.3 (81.2–109.5)	113.1 (59.8–213.9)	125.3 (82.6–190.1)	89.8 (54.9–146.7)	170.4 (100.4–289.2)
Post-vaccine	147.9 (79.0–165.1)	142.5 (118.7–171.2)	154.0 (132.1–179.6)	105.6 (65.9–169.1)	147.5 (86.6–251.3)	150.2 (81.9–275.3)	287.6 (178.2–464.4)
A/Swi H3N2							
Pre-vaccine	63.0 (58.1–68.4)	63.9 (55.2–73.9)	61.4 (54.9–68.6)	80.0 (32.5–196.9)	57.7 (41.3–80.8)	75.5 (53.3–107.1)	70.5 (46.9–105.9)
Post-vaccine	87.3 (79.0–96.5)	94.6 (79.5–112.7)	78.9 (69.8–89.1)	139.3 (23.9–812.7)	90.4 (60.4–135.3)	102.9 (52.8–200.6)	183.6 (133.4–252.9)
B/Phu Yamagata							
Pre-vaccine	73.2 (67.8–78.9)	74.8 (65.6–85.3)	71.4 (64.1–79.5)	67.3 (12.9–351.8)	76.8 (57.2–103.1)	75.5 (56.3–101.4)	75.1 (54.2–104.1)
Post-vaccine	77.4 (71.2–84.1)	75.1 (66.0–84.5)	73.6 (66.2–81.7)	114.5 (31.5–416.5)	86.8 (56.2–134.0)	102.9 (61.1–173.4)	110.2 (76.3–159.1)

Abbreviations: *DMARDs* disease modifying antirheumatic drugs, *bDMARDs* biological DMARDs

Data are GMT (95 % CI). ^a^ Numbers missing pre-vaccine and post-vaccine, respectively

**Table 3:** Percentage increase in antibody titers 28 days post vaccination

	All patients (*n* = 226) (missing^a^: 24/28)	bDMARDs monotherapy (*n* = 80) (missing^a^: 8/14)	bDMARDs + DMARDs (*n* = 110) (missing^a^: 13/11)	Rituximab (*n* = 5) (missing^a^: 1/0)	Systemic disorders (*n* = 18) (missing^a^: 1/1)	Other (*n* = 13) (missing^a^: 1/2)	Controls (*n* = 15) (missing^a^: 4/2)
A/Cal H1N1pdm09	62.7 (31.9–100.6)	44.9 (9.2–92.4)	82.2 (43.4–131.4)	2.2 (-45.6–129.2)	30.8 (-83.9–104.0)	108.2 (23.6–250.6)	74.2 (4.9–189.3)
A/Swi H3N2	74.9 (48.3–106.3)	89.9 (51.8–137.6)	64.3 (36.1–98.5)	63.3 (-86.9–206.9)	96.2 (38.5–177.9)	77.1 (17.7–166.5)	193.2 (96.8–336.5)
B/Phu Yamagata	12.3 (-98.3–28.4)	5.1 (-87.9–25.7)	6.7 (-91.8–23.9)	71.8 (5.1–180.9)	20.2 (-91.5–57.9)	37.2 (-99.6–88.9)	53.9 (12.5–110.5)

Abbreviations: *DMARDs* disease modifying antirheumatic drugs; *bDMARD* biological DMARDs

Data are % (95 % CI). ^a^ Numbers missing pre-vaccine and post-vaccine, respectively

A linear mixed model was used to estimate the increases from pre-vaccine to post-vaccine adjusted for the number of days between vaccination and post vaccination blood sampling. Percentage increase is reported at day 28 post vaccination

The estimated decreases in antibody levels per 30 days for the time interval 28–120 days post vaccination were as follows: A/Cal H1N1pdm09: -3.9 % (95 % CI -9.8–1.9); A/Swi H3N2: -7.6 % (95 % CI -12.3– -2.9); and B/Phu Yamagata: -1.0 % (95 % CI -4.8–2.8).

For all three influenza strains no associations were found between the increase in antibody level after vaccination and age, sex, duration of bDMARDs treatment, or time from injection of bDMARDs to vaccination (results not shown).

Seroprotection was evaluated for both pre-vaccine and post-vaccine samples at the cut offs for antibody titres of ≥ 40 and ≥ 110 (Table 4). Among all patients the pre-vaccine rates for seroprotection ≥ 40 were 93.1–97.0 % for all three strains. An increase in the proportions of patients being seroprotected after vaccination was found for A/Swi H3N2 whereas no increases were seen for A/Cal H1N1pdm09 and B/Phu Yamagata. For seroprotection ≥ 110 the pre-vaccine rates for all patients were 14.9 % – 43.6 % and for A/Cal H1N1pdm09 and A/Swi H3N2 this rose to 65.2 % and 31.3 % post-vaccine (both *p* ≤ 0.0001), respectively while there was no increase for B/Phu Yamagata.

**Table 4:** Seroprotection and seroconversion

	All patients (*n* = 226) % (n^a^/N^b^)	bDMARDs monotherapy (*n* = 80) % (n^a^/N^b^)	bDMARDs + DMARDs (*n* = 110) % (n^a^/N^b^)	Rituximab (*n* = 5) % (n^a^/N^b^)	Systemic disorders (except Rituximab) (*n* = 18) % (n^a^/N^b^)	Other (*n* = 13) % (n^a^/N^b^)	Controls (*n* = 15) % (n^a^/N^b^)
A/Cal H1N1pdm09							
Seroprotection							
Pre-vaccine 40	97.0 (196/202)	97.2 (70/72)	96.9 (94/97)	100 (4/4)	100 (17/17)	91.7 (11/12)	100 (11/11)
Post-vaccine 40	98.9 (196/198)	100 (66/66)	98.9 (98/99)	100 (5/5)	100 (17/17)	90.9 (10/11)	100 (13/13)
***p*-value**^c^	**0.10**	**-**	**0.16**	**-**	**-**	**1.0**	**-**
Pre-vaccine 110	43.6 (88/202)	54.2 (39/72)	35.1 (34/97)	50.0 (2/4)	47.1 (8/17)	41.7 (5/12)	72.7 (8/11)
Post-vaccine 110	65.2 (129/198)	63.6 (42/66)	66.7 (66/99)	40.0 (2/5)	58.8 (10/17)	81.8 (9/11)	84.6 (11/13)
***p*-value**^c^	< 0.0001*	**0.05**	< 0.0001*	**-**	**0.16**	**0.01**	**0.16**
Seroconversion	21.8 (38/174)	13.8 (8/58)	27.9 (24/86)	0 (0/4)	12.5 (2/16)	40.0 (4/10)	33.3 (3/9)
A/Swi H3N2							
Seroprotection							
Pre-vaccine 40	93.1 (188/202)	93.1 (67/72)	93.8 (91/97)	100 (4/4)	82.4 (14/17)	100 (12/12)	90.9 (10/11)
Post-vaccine 40	97.5 (193/198)	98.5 (65/66)	96.9 (96/99)	100 (5/5)	94.1 (16/17)	100 (11/11)	100 (13/13)
***p*-value**^c^	0.0016*	**0.045**	**0.045**	**-**	**0.16**	**-**	**-**
Pre-vaccine 110	14.9 (30/202)	15.3 (11/72)	13.4 (13/97)	25.0 (1/4)	11.8 (2/17)	25.0 (3/12)	18.2 (2/11)
Post-vaccine 110	31.3 (62/198)	34.9 (23/66)	28.3 (28/99)	40.0 (2/5)	29.4 (5/17)	36.4 (4/11)	84.6 (11/13)
***p*-value**^c^	< 0.0001*	0.006*	0.0002*	**-**	**0.16**	**0.16**	**0.01**
Seroconversion	18.4 (32/174)	15.5 (9/58)	19.8 (17/86)	25.0 (1/4)	18.8 (3/16)	20.0 (2/10)	66.7 (6/9)
B/Phu Yamagata							
Seroprotection							
Pre-vaccine 40	97.0 (196/202)	95.8 (69/72)	97.9 (95/97)	75.0 (3/4)	100 (17/17)	100 (12/12)	100 (11/11)
Post-vaccine 40	100 (198/198)	100 (66/66)	100 (99/99)	100 (5/5)	100 (17/17)	100 (11/11)	100 (13/13)
***p*-value**^c^	**-**	**-**	**-**	**-**	**-**	**-**	**-**
Pre-vaccine 110	17.8 (36/202)	18.1 (13/72)	14.4 (14/97)	50.0 (2/4)	29.4 (5/17)	16.7 (2/12)	18.2 (2/11)
Post-vaccine 110	19.7 (39/198)	15.2 (10/66)	17.2 (17/99)	40.0 (2/5)	35.3 (6/17)	36.4 (4/11)	53.9 (7/13)
***p*-value**^c^	**0.35**	**0.71**	**0.26**	**0.32**	**0.16**	**0.32**	**0.045**
Seroconversion	6.9 (12/174)	5.2 (3/58)	5.8 (5/86)	50.0 (2/4)	6.3 (1/16)	10.0 (1/10)	22.2 (29)

Abbreviations: *DMARDs* disease modifying antirheumatic drugs; *bDMARDs* biological DMARDs

^a^For seroprotection, number of patients with antibody level above threshold. For seroconversion, number fulfilling the criteria for seroconversion. ^b^Total number of patients with available blood sample

^c^The proportion of children increasing to a protective level of antibodies was analysed using McNemars test. Only children with both pre-vaccine and post-vaccine blood samples were included in the analyses. The calculation of the p-value was not possible when one of the proportions was 100 %

*Only *p*-values < 0.007 = 0.05/7 were considered statistically significant after Bonferroni correction for multiple testing for each outcome and each cut off

**Fig. 2:**
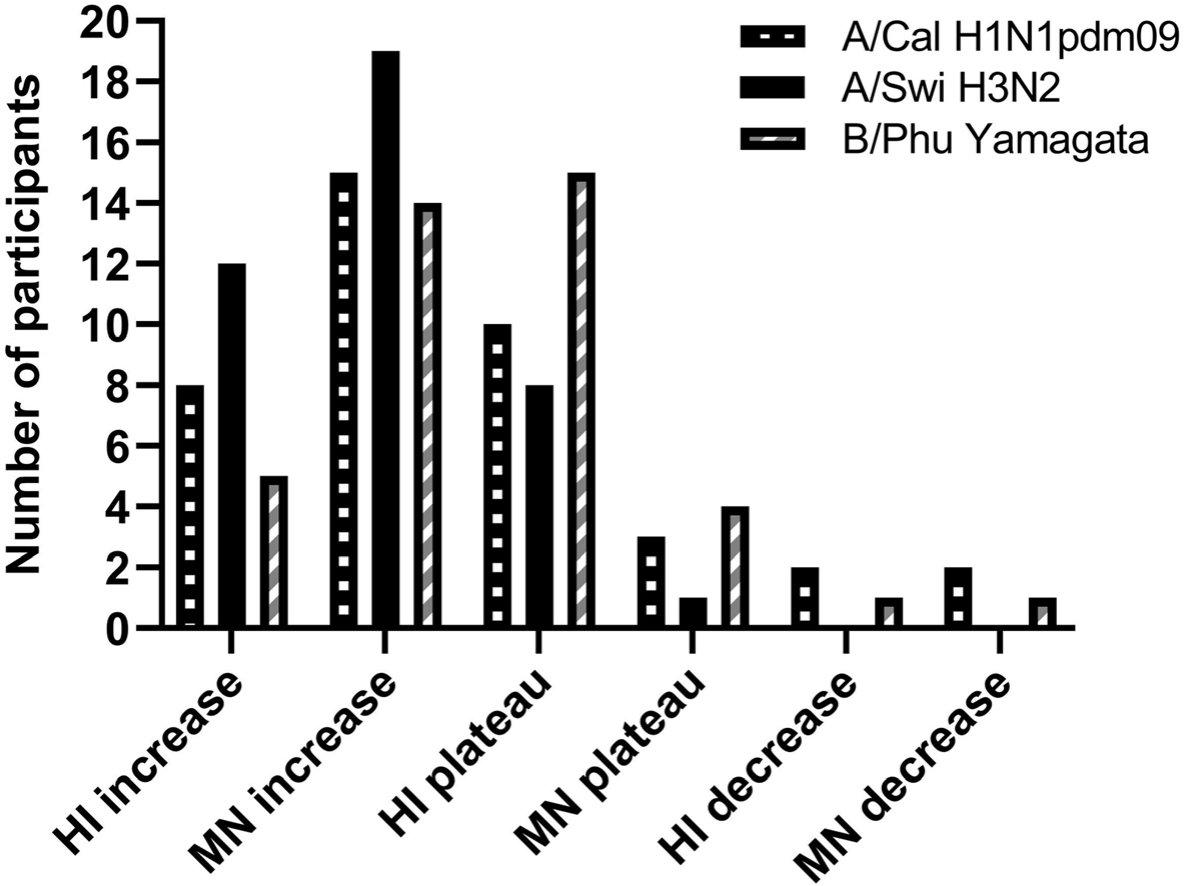
Response to vaccination using haemagglutination inhibition assay and microneutralisation assay . Abbreviations: HI: haemagglutination inhibition; MN: microneutralisation. A selection of samples was both tested for the presence of antibodies using the HI assay and for functional antibodies by MN. From the pre-vaccine sample to the post-vaccine sample the antibody titre was shown to increase, decrease or remain at a plateau

The MN assay was used for reanalysing a subset of the blood samples (11 patients and 9 controls) to investigate the performance of the antibodies in this functional assay. The results showed that participants having an increase in antibody titres in the HI assay also increased with the MN assay (Fig. 2). Furthermore, we found that more participants increased in the MN assay compared to the HI assay.

Flu-like illness was registered in 42 patients of whom 5 experienced 2 episodes, compared to no episodes among controls. The frequencies of symptoms registered in 96 % of the episodes: Malaise 87 %, sore throat 76 %, head ache 76 %, fever 73 %, cough 63 %, sore muscles 53 %, and stomach ache 44 %. None of the patients were tested positive for any of the influenza strains included in the vaccine but 12 (26 %) were tested positive for influenza B Victoria.

## Discussion

In this prospective multicentre cohort study, we investigated the response to vaccination against seasonal influenza 2015–2016 in children with rheumatic diseases and immunologically healthy controls; all patient groups and controls increased in antibody titres after vaccination.

The pre-vaccine GMTs for A/Swi H3N2 and B/Phu Yamagata were about 70 for both patients and controls, whereas for A/Cal H1N1pdm09 pre-vaccine GMT was about 100 for patients and 170 for controls. In this study we had no information about previous vaccinations against influenza but due to medical treatment and disease some children had been vaccinated before. Previous infections by circulating virus strains could affect these titres, however A/Cal H1N1pdm09 had been included in the seasonal vaccine in the years preceding our study and this could explain the higher pre-vaccine titre against this strain [14]. In contrast, A/Swi H3N2 and B/Phu Yamagata were both introduced in the 2015–2016 vaccine and this may explain the lower pre-vaccine titres [21].

No differences in the percentage increase in antibody titres after vaccination between patient groups and controls were found. However, when combining all the patients across groups into one group and comparing antibody increase between these and controls we found a lower response for patients to A/Swi H3N2, and a tendency to a lower response for B/Phu Yamagata. It is possible that we would have found a difference in the initial comparisons between groups if the number of participants in each group had been larger.

More than 90 % of patients and controls were already protected pre-vaccine against the three influenza strains using the cut-off for seroprotection ≥ 40. Consequently, the proportion of patients reaching this protective level after vaccination was only significant for A/Swi H3N2.

The proportion of children protected pre-vaccine was substantially lower using the cut-off for seroprotection ≥ 110 and only few groups had a significant proportion of patients achieving this level after vaccination. Since most participants in our study were older than 6 years and no clearly defined cut off for seroprotection in children above 6 years exists, it is therefore uncertain whether we should assume that the patients were mainly protected or unprotected. A likely explanation for patients not reaching seroprotection ≥ 110 could be immunosuppression as we found that for all three strains the percentages of controls protected after vaccination were higher for controls than patients. However, the crude numbers for antibody titres were used for estimation of seroprotection and direct comparisons between groups were not performed. As the post-vaccine blood samples were taken later for patients than controls, and antibodies were shown to decline beyond day 28 post vaccination, some patients might have been misclassified as being unprotected compared to controls who had the post-vaccine blood sample taken earlier.

Previous studies in children with rheumatic diseases have focused on the seroprotection ≥ 40 cut off [5, 7, 8]. As our results showed that the proportions of children being protected after vaccination were very different depending on which cut off was used, it emphasizes the need for defining a better correlate for protection in children above 6 years in future clinical studies.

We investigated the differences in antibody response depending on immunosuppression, and the lowest increase was expected for the patients receiving Rituximab. But as described above, no differences were found between the patient groups, and even when looking at the percentage increase in antibody titres and proportions of patients achieving seroprotection, there were not a clear tendency for any group to have a better or worse response to all three influenza strains than the remaining groups.

Previous studies of response to seasonal influenza vaccine among patients with rheumatic diseases are not comparable due to differences in rheumatic disease and/or laboratory method and/or influenza virus strain but the overall findings are in line with our results [6–8].

As our results showed a limited increase in antibody titres, we chose to analyse the functionality of antibodies in a sub-set of samples using the MN assay. We found that participants having increasing antibody titres using the HI assay also increased using the MN assay. Furthermore, a higher number of participants had increasing antibody titres using the MN assay compared to the HI assay indicating that the MN assay was more sensitive. This is in line with previous findings [22] and it strengthens the assumption that our results from the HI assays are a reliable correlate for antibody response to the influenza vaccine in our cohort.

The population were primarily infected by A/Cal H1N1pdm09 (51 %), or influenza B Victoria (44 %) in the 2015–2016 influenza season in Denmark [21]. The influenza B Victoria strain was not included in the vaccine [21]. Since patients with influenza-like illness only tested positive for influenza B Victoria, it was not possible to investigate the relation between antibody levels and clinical infection caused by the three strains included in the vaccine. The influenza A/Cal H1N1pdm09 strain was circulating but no clinical cases were seen in our cohort possible due to protective antibody levels.

A strength for this study is the prospectively inclusion from three geographical different centres, and we assume that it is representative for paediatric patients with rheumatic disease who are vaccinated against seasonal influenza due to immunosuppression. About one third of all Danish JIA patients treated with bDMARDs participated in this study, thereby contributing with important knowledge from a larger group of patients compared to previous studies [7–9].

The study also has some limitations. Only a minor number of controls were included and thus we were not able to conduct the intended gender and age-matched study design. We intended to include 139 patients and 69 controls for comparisons, but this was not achieved due to difficulties in recruiting and it limits our ability to find a difference between the groups.For the completed participants 20 % had a missing blood sample either pre-vaccine or post-vaccine. The reason for missing samples was not registered but we assume that they were missing at random. Therefore, we chose a statistical model where all patients contributed even when either the pre-vaccine or post-vaccine sample was missing. Furthermore, the patients in our study had the post-vaccine blood sample taken later than controls, and due to this imbalance, we adjusted for days from vaccination to post-vaccine blood sampling in the analyses. Lastly, we grouped the patients based on medical treatment or disease, but the results are weak due to small numbers.

## Conclusions

In conclusion, children with rheumatic disease and healthy controls increase in antibody titres after influenza immunization. Depending on the cut-off for seroprotection it remains, nevertheless, unclear whether a protective level is achieved. However, none of the patients and healthy controls were tested positive for any of the influenza strains included in the vaccine. The results suggest that influenza vaccination provides protection in immunocompromised children with rheumatic disease, although possibly less so than in healthy children.

Our findings emphasize the need for defining a better correlate for protection in children in future clinical studies and lastly, time from vaccination to post-vaccine blood sampling was found to be an important confounder so future studies should take this into account.

## Data Availability

The datasets generated and analysed during the current study are not publicly available. Consent for making the datasets publicly available was not obtained.

## Abbreviations

A/Cal H1N1pdm09: A/California/07/2009-like (H1N1)pdm09
A/Swi H3N2: A/Switzerland/9715293/2013-like (H3N2)
B/Phu Yamagata: B/Phuket/3073/2013-like (Yamagata-lineage)
bDMARDs: Biological disease modifying antirheumatic drugs
DMARDs: Disease modifying antirheumatic drugs
GMT: Geometric means titre
HI: Haemagglutination inhibition
JIA: Juvenile idiopathic arthritis
MN: Microneutralisation
